# The Current and Future Distribution Areas of the Rare and Endangered Fern *Cibotium barometz* (Cibotiaceae) in Guizhou, China, Based on the MaxEnt Model and ArcGIS

**DOI:** 10.1002/ece3.74203

**Published:** 2026-08-26

**Authors:** Fulin Yan, Songsong Wu, Wenxi Xiang, Qingwen Sun, Shenghua Wei

**Affiliations:** ^1^ Guizhou University of Traditional Chinese Medicine Guiyang China

**Keywords:** ArcGIS, *Cibotium barometz* (L.) J. Sm, climate adaptability, MaxEnt model, suitable habitat zoning

## Abstract

Climate change poses an increasing threat to rare medicinal plants, yet the response mechanisms of many endangered species remain poorly understood. *Cibotium barometz* (L.) J. Sm., listed in the Convention on International Trade in Endangered Species of Wild Fauna and Flora (CITES), is a typical example of this conservation challenge due to its overexploitation for traditional medicinal use in China. In this study, the MaxEnt model and ArcGIS software were used to predict the potential suitable habitats for *C. barometz* in China under current and future climate scenarios (from SSP1‐2.6 to SSP5‐8.5). The results showed that precipitation of the driest quarter (bio17) contributed the most to model construction, with an optimal precipitation range of 50–150 mm. The species distribution model indicated that under current climatic conditions, the national high‐suitability zone encompasses 24,410 km^2^, predominantly located in Guangdong, Guangxi, and southwestern Guizhou. Under future climate scenarios, the suitable habitat area of *C. barometz* shows an increasing trend under the low‐emission pathway (SSP1‐2.6), but faces a continuous decline risk under medium‐emission pathways (SSP2‐4.5 and SSP3‐7.0). Under the high‐emission scenario (SSP5‐8.5), the area of high‐suitability habitat expands to 279,800 km^2^, with its centroid shifting significantly southward by 148.95 km. Consequently, this study identifies priority areas for in situ conservation (southwestern Guizhou and southern Guangdong) and key regions for ex situ conservation (central Guizhou), providing a crucial reference for the sustainable utilization of this endangered medicinal fern.

## Graphical abstract

Using MaxEnt and ArcGIS, we modeled the distribution of the endangered medicinal fern *Cibotium barometz* under current and future climate scenarios (SSP1‐2.6 to SSP5‐8.5). Precipitation of the Driest Quarter (49.9% contribution) was the key determinant, and projections indicate that under the high‐emission pathway (SSP5‐8.5), the high‐suitability habitat could expand to 279,800 km^2^ by the 2090s, accompanied by a pronounced southward centroid shift. These findings provide critical insights for conservation planning, including the establishment of reserves and ex‐situ conservation strategies.

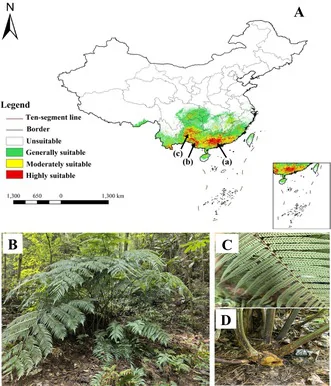

## 1 Introduction

Climate is a key determinant of species' geographical distribution and ecological adaptability (Sánchez‐Castro et al. 2024). In recent decades, against the backdrop of global climate warming, the suitable distribution areas of plant species have generally migrated, which has a profound impact on biodiversity and ecosystem functions (Jing et al. 2022). Therefore, simulating the dynamic response of potential species distribution areas to climate change is an important basis for assessing the risk of future biodiversity changes and formulating scientific protection strategies.

Niche model is an important tool for simulating the potential distribution of species in ecology. By analyzing the known occurrence data of species and relevant environmental variables, specific algorithms can be applied to construct relationship models between species distribution and environmental factors, thereby assessing species' niche requirements. Major approaches include Ecological Niche Factor Analysis (ENFA), Genetic Algorithm for Rule‐Set Production (GARP), Bioclim, Domain, and Maximum Entropy (MaxEnt), among others (Vasconcelos et al. 2024; Cao et al. 2010). Compared with other models, the MaxEnt model has higher prediction accuracy and stability (Li et al. 2021). The model has been applied to the ecological suitability zoning of various medicinal plants, including *Epimedium* (Lai, Lai, et al. 2025; Lai, Cui, et al. 2025), *Stemona* (Huangfu et al. 2024), and *Scutellaria baicalensis* (He et al. 2023).

**FIGURE 1:**
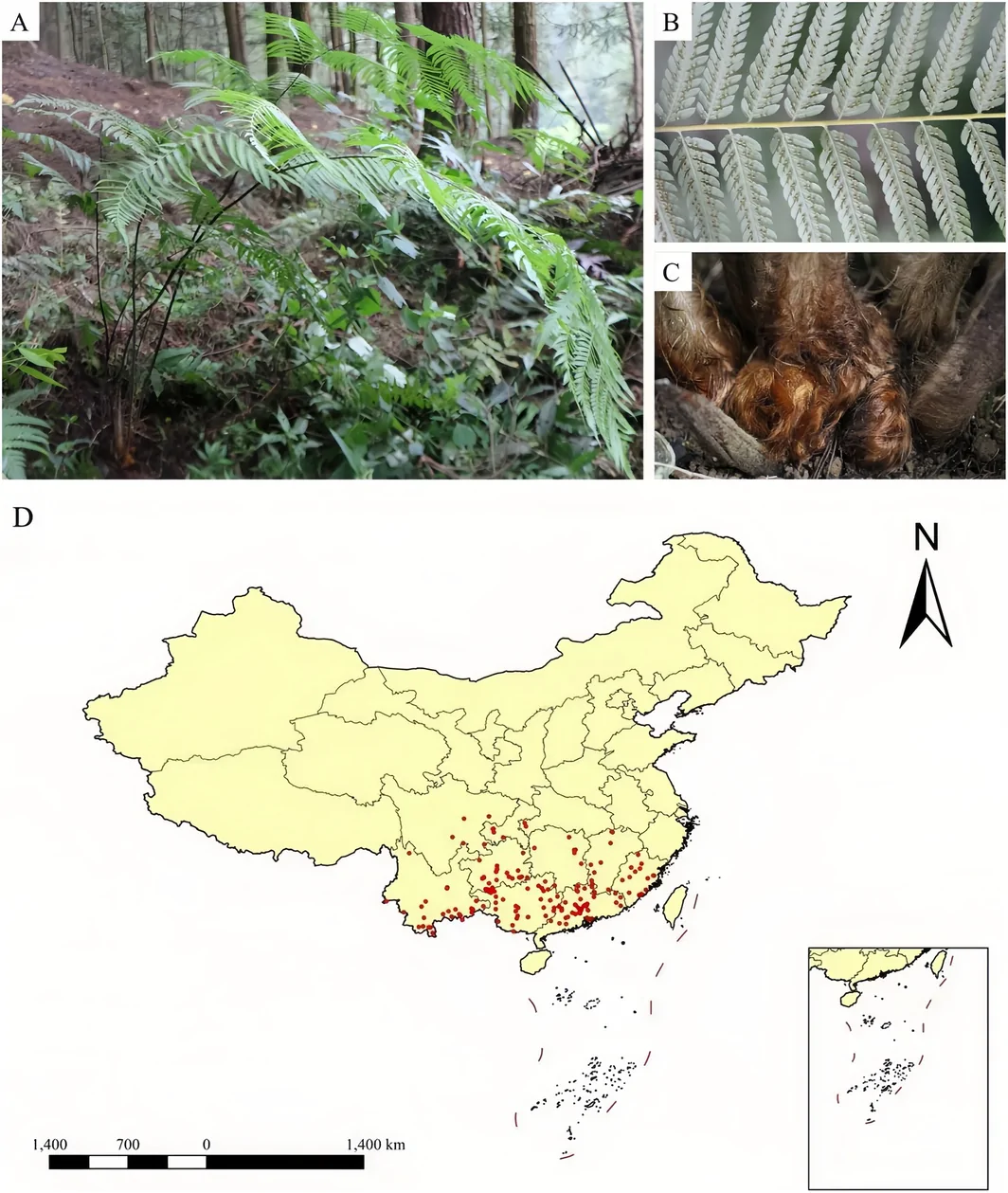
Photographs (A–C) and occurrence records (D) of *C. barometz* in China. All photographs were taken by Songsong Wu. This map is drawn based on the standard map from the Map Technology Review Center of the Ministry of Natural Resources [Review No.: GS (2024)1158]. The base map boundaries are unmodified. The same applies below.

*Cibotium barometz* (L.) is a perennial large tree‐like terrestrial fern belonging to *Cibotium* Kaulf., Cibotiaceae. Its rhizome is the only source of traditional Chinese medicine “Cibotii Rhizoma.” It has the effects of dispelling rheumatism, tonifying liver and kidney, and strengthening waist and knees. It can be used as a strong agent (Editorial Committee of Flora of China, Chinese Academy of Sciences 2004; National Pharmacopoeia Committee 2020). A cluster of large pinnate fronds arises from the apex of the rhizome (Figure 1A). On the abaxial surface of the fertile fronds, transversely elongated sori are covered by a hard, bivalvate indusium. At maturity, the indusium dehisces like a mussel shell, exposing the sori (Figure 1B). The petiole bases are densely covered with golden trichomes (Figure 1C). It has unique morphology and can be used as a hemostatic agent, which has important ornamental and medicinal value (Ye et al. 2020). *C. barometz* is an indicator plant for tropical and subtropical acidic soils in southern China. It is mainly distributed in Yunnan, Guizhou, southern Sichuan, Guangdong, Guangxi and Fujian provinces and regions in China (Figure 1D). It is also distributed in India, Myanmar, Thailand, Vietnam, Laos, Cambodia, Indonesia, the Philippines and other regions (Yu et al. 2023). Despite the wide distribution of *C. barometz* , the amount of wild resources is decreasing due to factors such as increased habitat destruction and human exploitation. It has been listed as a national second‐class protected plant in China (i.e., species with relatively small wild populations, narrow distribution, or significant economic/scientific value; collection and trade are strictly regulated under national law) and included in Appendix II of the Convention on International Trade in Endangered Species of Wild Fauna and Flora (CITES) (Pang et al. 2026).

As a relic fern species with highly specialized ecological requirements, *C. barometz* occupies a remarkably narrow ecological niche. Its survival and reproduction are critically dependent on specific microclimatic conditions: it is strictly confined to warm (optimal mean annual temperature 16°C–22°C), humid (relative humidity about 70%–80%), and semi‐shaded to deeply shaded understories within evergreen broad‐leaved forests or ravine slopes. It exhibits pronounced intolerance to frost (likely lethal below 0°C–5°C), drought stress, and direct sunlight exposure, which can rapidly lead to desiccation and mortality (Chen et al. 2022a). Beyond climatic variables, the species' distribution is tightly constrained by edaphic and geological factors. It requires well‐drained, acidic, organically rich soils, and is closely associated with acidic soil patches (e.g., yellow and red soils) and acidified microhabitats within southern China's karst landscapes (Wu et al. 2024). This intricate dependency on stable temperatures, high humidity, specific light regimes, and particular substrates highlights the species' ecological specialization and inherent vulnerability. This intricate dependence on a combination of stable temperature, high humidity, specific light levels, and specialized substrates underscores its ecological specialization and vulnerability. Thus, the stringent and interconnected requirements make *C. barometz* exceptionally vulnerable to environmental fluctuations, serving as a sensitive indicator of intact forest ecosystems and stable microclimates within the subtropical zone. In recent years, the related research on *C. barometz* has mainly focused on spore germination and gametophyte development (Peng 2013; Pu 2015), tissue culture (Yu et al. 2024; Yang et al. 2026), DNA barcoding (Yin et al. 2023; Zhao et al. 2025) and pharmacological activity (Chen et al. 2022b; Sun et al. 2025), and there are only a few reports in ecology (Chen et al. 2025b; Jiang et al. 2026).

Therefore, in this study, we used the MaxEnt model and ArcGIS technology to predict the suitable habitat distribution of *C. barometz* under current and future climate scenarios. The objective was to delineate priority conservation areas for wild populations and identify optimal cultivation zones suitable for managed growth, thereby providing a theoretical basis for the conservation of wild resources, strategic planning of cultivation practices, and the sustainable utilization of *C. barometz* .

## 2 Materials and Methods

### 2.1 Acquisition and Processing of Geographical Distribution Data of *C. barometz*

Geographical occurrence records for *C. barometz* were sourced from the Global Biodiversity Information Facility (GBIF; http://www.gbif.org) and China's National Specimen Information Infrastructure (NSII; http://www.nsii.org.cn), comprising 1654 initial records supplemented by 16 field‐surveyed occurrences. To prevent overfitting of experimental results caused by data redundancy, data with duplicate latitude and longitude or unclear coordinates were initially filtered out. The retained data were imported into ArcGIS 10.8 via Excel, with occurrence records converted to equal‐area 1 km × 1 km grid cells matching the resolution of ecological variables. A single occurrence was retained per grid cell before exporting to CSV format (Du et al. 2022), yielding 191 refined occurrence records for current habitat analysis, as shown in Figure 1D.

### 2.2 Environmental Variable Data and Processing

This study incorporated 37 ecological variables, including 19 climatic factors (World Clim bioclimatic variable bio1‐bio19), 3 topographic factors (altitude, aspect, slope), and 15 soil factors (soil pH, organic carbon content, cation exchange capacity, etc., in Table 1). Climate data were obtained from World Clim (http://www.worldclim.org/); current http://www.worldclim.org/ soil variables originated from the National Cryosphere Desert Data Center (http://ncdc.ac.cn/). The resolution of all variables is unified to 30 arc‐seconds and reformatted in ArcGIS 10.8 for subsequent analysis. Current climate adopts the average value from 1970 to 2000, and future climate projections for the 2030s, 2050s, 2070s, and 2090s utilized the CMIP6‐era BCC‐CSM2‐MR model from China's National Climate Center—featuring enhanced atmospheric and terrestrial resolution that improves topographic representation and better simulates orographic precipitation and local temperature distributions (Ding et al. 2024). Four representative SSP scenarios were selected: SSP126, SSP245, SSP370, and SSP585.

**TABLE 1:** Information on ecological factors.

Abbreviation	Ecological factor	Abbreviation	Ecological factor
bio1	Annual mean temperature	alt	Elevation
bio2	Mean diurnal range	asp	Aspect
bio3	Isothermality	slo	Slope
bio4	Temperature seasonality	t_bs	Base saturation
bio5	Max temperature of warmest month	t_caco3	Carbonate or lime content
bio6	Min temperature of coldest month	t_cec_clay	Cation exchange capacity of clay fraction
bio7	Temperature annual range	t_cec_soil	Soil cation exchange capacity
bio8	Mean temperature of wettest quarter	t__clay	Clay content
bio9	Mean temperature of driest quarter	t_ece	Electrical conductivity
bio10	Mean temperature of warmest quarter	t_esp	Exchangeable sodium percentage
bio11	Mean temperature of coldest quarter	t_gravel	Gravel volume percentage
bio12	Annual precipitation	t_oc	Organic carbon content
bio13	Precipitation of wettest month	t_ph_h2o	Soil pH (H_2_O)
bio14	Precipitation of driest month	t_ref_bulk	Soil bulk density (Reference)
bio15	Precipitation seasonality	t_sand	Sand content
bio16	Precipitation of wettest quarter	t_silt	Silt content
bio17	Precipitation of driest quarter	t_teb	Total exchangeable bases
bio18	Precipitation of warmest quarter	t_usda_tex	USDA soil texture classification
bio19	Precipitation of coldest quarter		

### 2.3 Screening of Ecological Factors

Distribution point data and the clipped 37 ecological variables were imported into ArcGIS 10.8. Using the Spatial Analyst toolbox, values were extracted to point locations via the “Extract Multi Values to Points” tool. The output was then imported into SPSS 26.0 for bivariate correlation analysis. Environmental variables exhibiting absolute correlation coefficients |r| > 0.8 underwent pairwise evaluation, with one stochastically retained variable per collinear pair for subsequent modeling in MaxEnt 3.4.4.

### 2.4 Construction of the MaxEnt Model

The “ *C. barometz* .csv” distribution data and filtered ecological variables were imported into MaxEnt 3.4.4 for predictive modeling. Parameters were configured as follows (Huang et al. 2024): Random test percentage = 25%; Regularization multiplier = 1; Maximum background points = 10,000; Replicates = 10; Maximum iterations = 5000; Convergence threshold = 10^−5^; Replicated run type = Bootstrap. Jackknife validation and response curve generation were enabled, with other parameters set to default values.

### 2.5 Establishment of the MaxEnt Model

The Area Under the Curve (AUC) value represents the area beneath the Receiver Operating Characteristic (ROC) curve, serving as a key metric for evaluating model performance in species distribution prediction. Higher AUC values indicate greater predictive accuracy and reliability (Li et al. 2021). Model interpretation follows established thresholds: 0.9 ≤ AUC < 1 denotes excellent predictive performance; 0.8 ≤ AUC < 0.9 signifies good accuracy; 0.7 ≤ AUC < 0.8 suggests acceptable discrimination; 0.6 ≤ AUC < 0.7 indicates poor performance; and 0.5 ≤ AUC < 0.6 constitutes model failure (Wang et al. 2020).

### 2.6 Ecological Suitability Level Classification

The averaged output from 10 MaxEnt model replicates was imported into ArcGIS and exported as an ASCII raster layer. Based on presence probability (*p*‐value), ecological suitability zones for *C. barometz* were classified into four tiers: high‐suitability (*p* > 0.5), medium‐suitability (0.3 ≤ *p* ≤ 0.5), low‐suitability (0.1 ≤ *p* < 0.3), and non‐suitable areas (*p* < 0.1) (Ji et al. 2023). Furthermore, leveraging the national ecological suitability results, provincial‐scale suitability predictions were refined specifically for Guizhou Province.

### 2.7 Centroid Shift

Centroid shift refers to the spatial displacement of the geographic center (the geometric centroid of the distribution range) of species distribution over time or environmental changes. It is often used to quantify the response of species to climate change (Chen et al. 2025a). In this study, the fusion operation in the data management tool of ArcGIS 10.8 software was used to fuse the units with a value of 1 in each period, that is, the suitable area unit, into a vector particle. The “average center,” “merge,” and “point set transfer line” tools in ArcGIS were used to draw the centroid site and migration trajectory of the suitable area. (Lai, Lai, et al. 2025; Lai, Cui, et al. 2025; He et al. 2025).

## 3 Results

### 3.1 Correlation of Ecological Factors for *C. barometz* and Assessment of MaxEnt Model Predictive Ability

**FIGURE 2:**
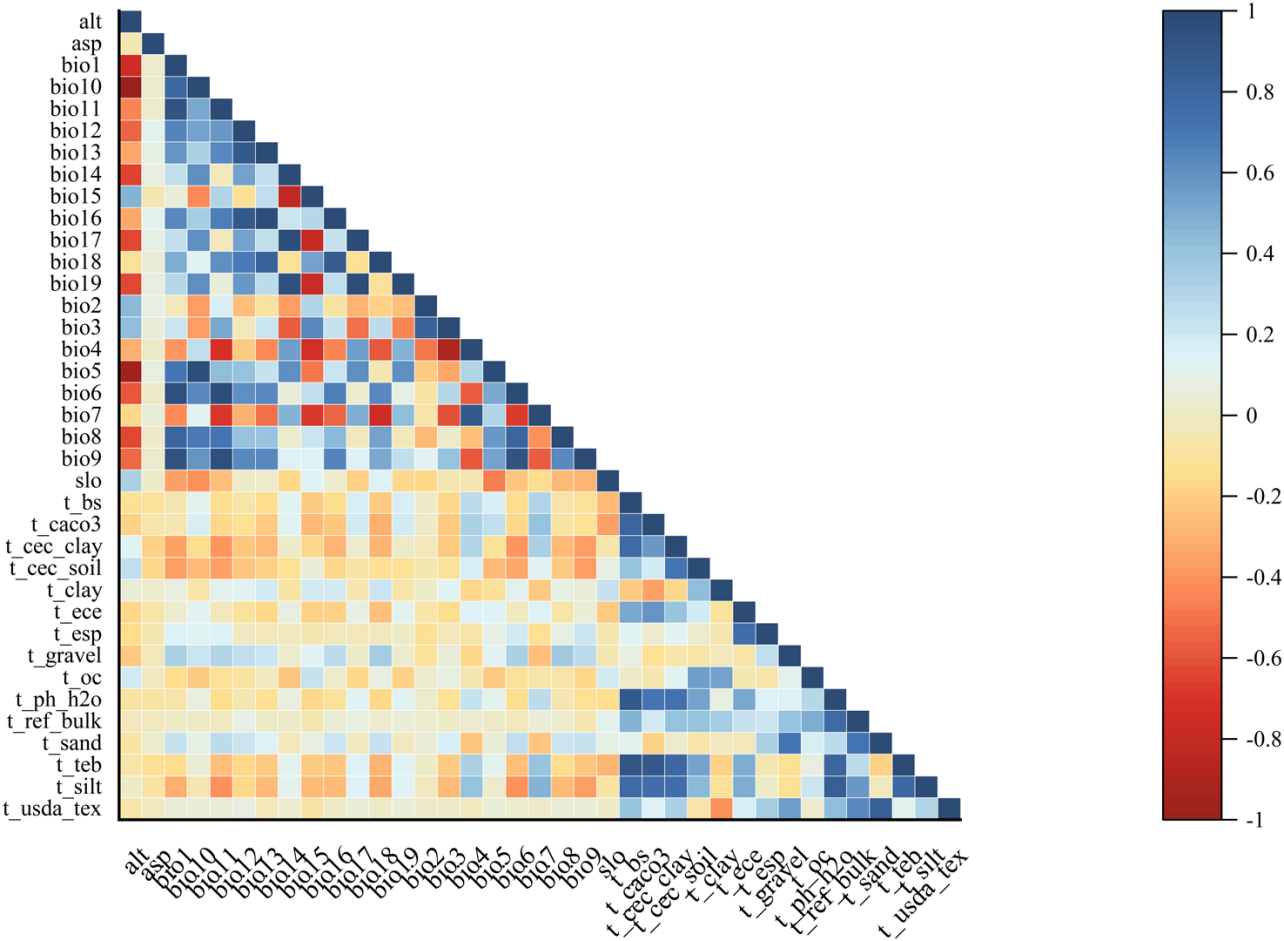
Correlation analysis of ecological factors for *C. barometz*.

**FIGURE 3:**
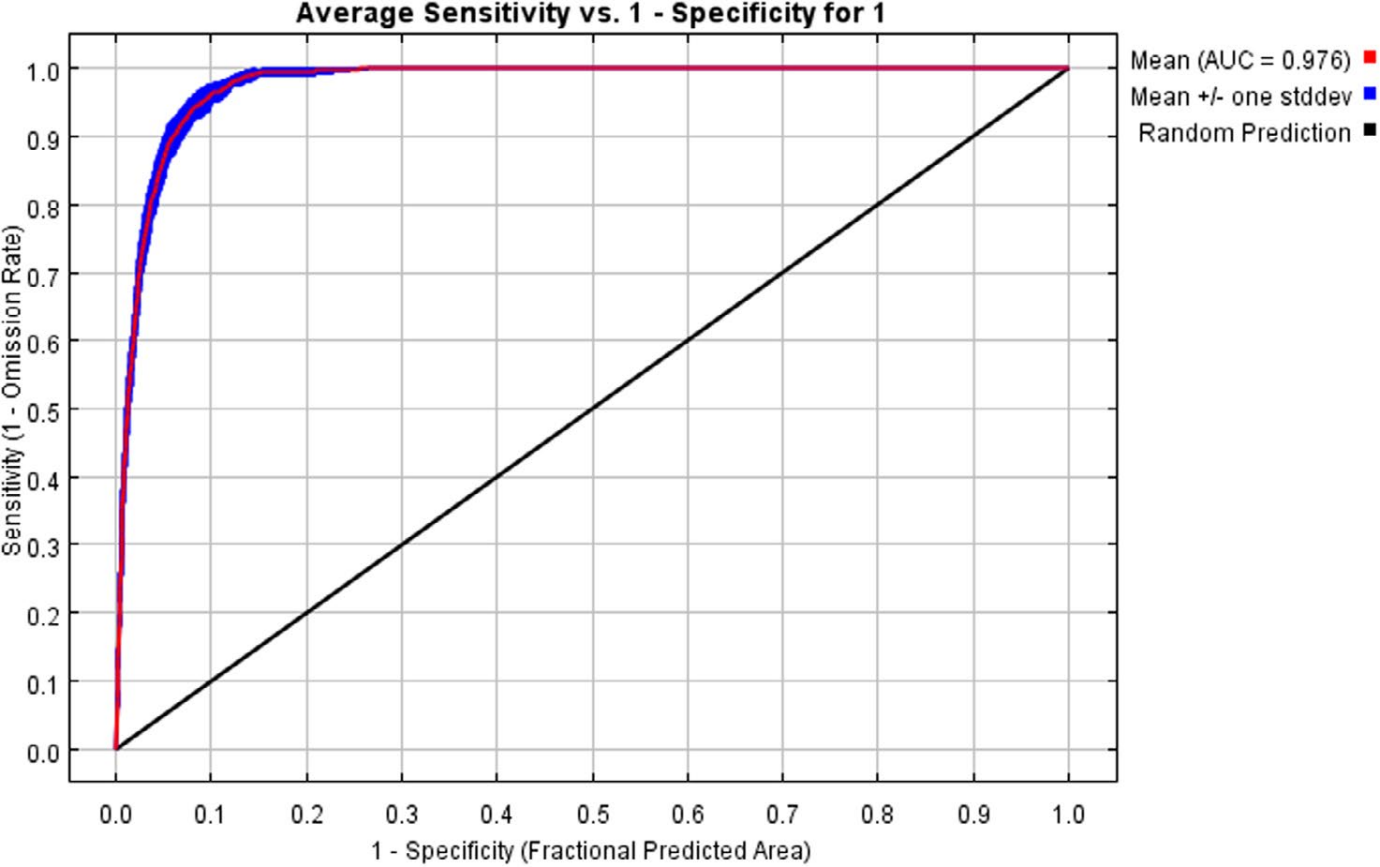
ROC curve of *C. barometz* prediction model.

Correlation analysis revealed that 5 of the 37 ecological factors exhibited absolute correlation coefficients |r| > 0.8: bio 6, bio 9, bio 11, bio 12, and t_bs. (Figure 2). To avoid model overfitting and unstable coefficient estimates caused by multicollinearity, these five variables were excluded from the final model because the environmental information they represent was already adequately captured by other retained variables (e.g., temperature‐related information was represented by bio4 and bio8; precipitation‐related information by bio15 and bio17; and t_bs was redundant with bio4). Thus, excluding these five variables removed redundant information without substantially compromising the independent explanatory power of the environmental dataset. The MaxEnt model demonstrated high predictive reliability for *C. barometz* , with a mean AUC value of 0.976 across 10 replicate runs (Figure 3), indicating excellent accuracy in delineating ecological suitability zones.

### 3.2 Current Suitable Habitat and Area Statistics for *C. barometz*

**FIGURE 4:**
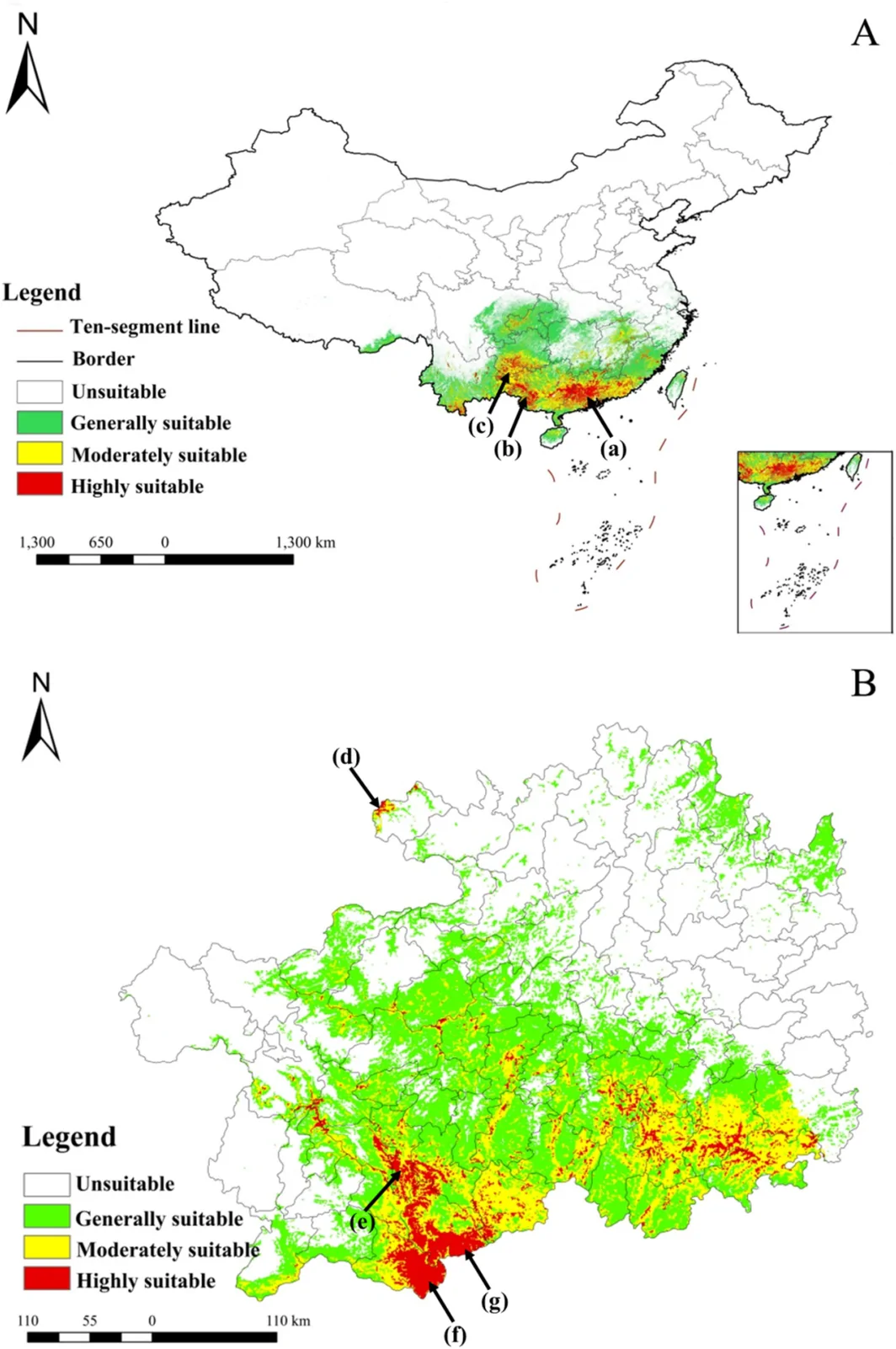
A: Current national distribution of suitable habitats for *C. barometz* ; (a) Guangdong; (b) Guangxi; (c) Guizhou; B: Current distribution of suitable habitats for *C. barometz* within Guizhou Province; (d) Chishui City; (e) Zhenning Buyi and Miao Autonomous County; (f) Ceheng County; (g) Wangmo County. Black arrows indicate the high suitable areas for *C. barometz* under current conditions.

At present, the suitable areas of *C.barometz* are distributed in southern China, including Guangdong, Guangxi, Jiangxi, Guizhou, northwestern Hunan, southern Yunnan, northern Hainan and other places (Figure 4A). Among them, the highly suitable area is 24,410 km^2^, accounting for 1.5% of the national area. It is mainly distributed in most areas of Guangdong, western and eastern Guangxi, southwestern Guizhou (has been marked with a black arrow) and other places. The above areas are mainly mountainous and hilly terrains, and most of them are karst landforms. The winter is mild, the annual temperature difference is small, the precipitation resources are abundant, and it is suitable for the growth of golden poodle ridge. The area of medium and low suitable areas is 180,596 km^2^, accounting for 11.07% of the total area of the country. It mainly spreads around Guizhou, Guangxi and Guangdong in a ring. The non‐suitable area is 1,425,545 km^2^, accounting for 87.43% of the national area. It is mainly distributed in alpine or arid areas such as Tibet, Qinghai, Xinjiang and Northeast China.

Currently, the suitable areas of *C. barometz* in Guizhou Province are mainly distributed in Qiandongnan Miao and Dong Autonomous Prefecture, Qiannan Buyi and Miao Autonomous Prefecture, Guiyang City, Anshun City and Zunyi Chishui (Figure 4B). The high suitable area is 6402 km^2^, which is mainly distributed in the southwest of Chishui City, most of the eastern part of Ceheng County, the south of Wangmo County, and the south of Zhenning Buyi and Miao Autonomous County (has been marked with a black arrow). The above areas are mainly middle and low mountains, karst and Danxia landforms coexist, and belong to subtropical humid monsoon climate. The annual average temperature is about 19°C. The precipitation is abundant and mostly concentrated in summer, and extreme cold weather is rare in winter. In summary, the suitable habitat areas for *C. barometz* are mainly located in regions with a subtropical monsoon climate, characterized by pronounced karst topography, such as mountains and hills, abundant precipitation, and relatively minor temperature fluctuations.

### 3.3 Prediction of Suitable Habitat Change Trends Under Future Climate Scenarios for *C. barometz*

**FIGURE 5:**
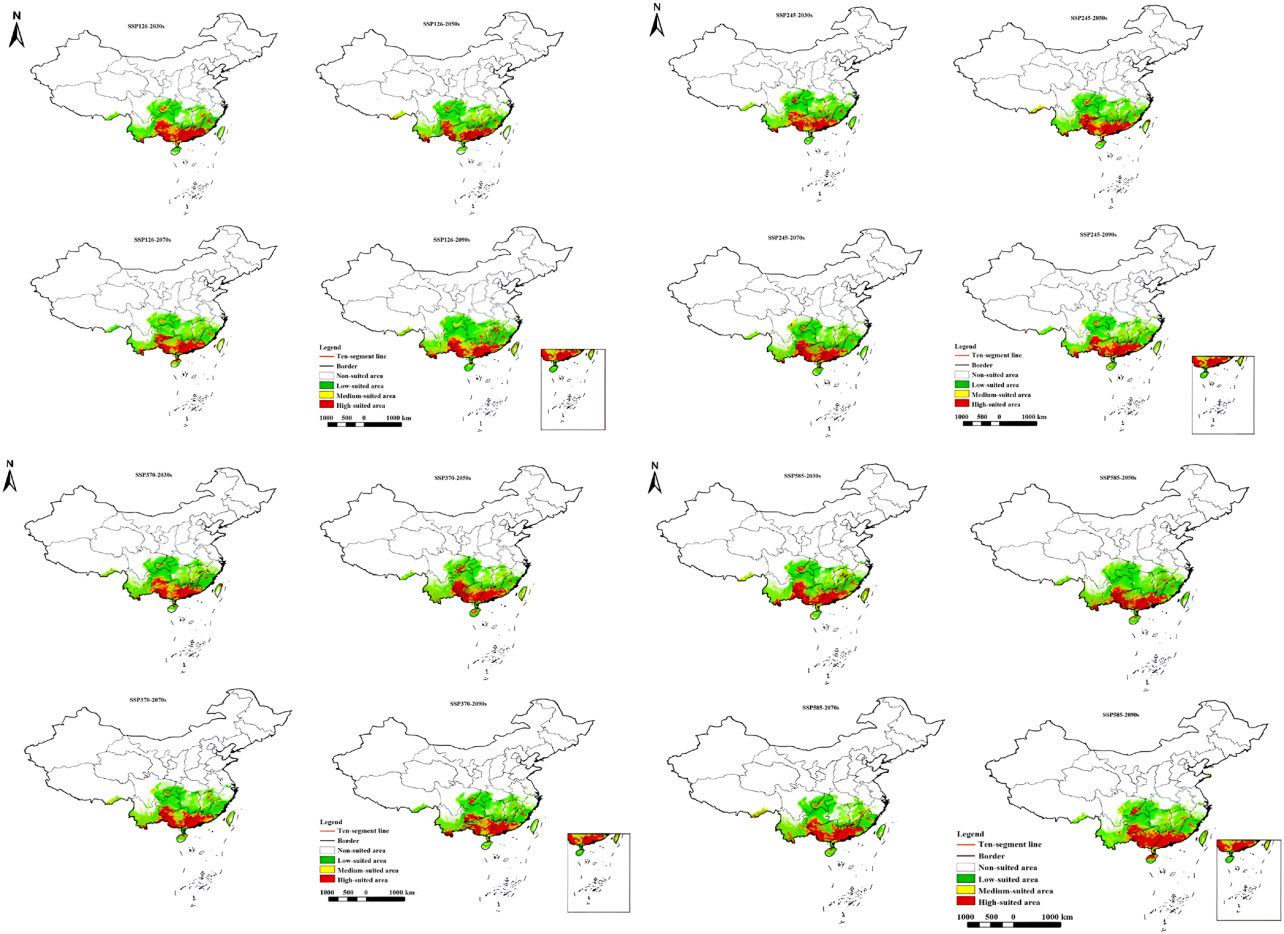
Distribution of suitable habitats for *C. barometz* under future climate scenarios.

The distribution of suitable habitat areas for *C. barometz* under future climate scenarios and its changing trends are shown in Figure 5, with changes in suitable area size detailed in Table 2. The results indicate that under relatively mild climate scenarios, the high‐suitability zone shows a tendency to expand slightly toward higher elevations and northern regions. This may be due to continuously rising temperatures, with some low‐suitability areas in low‐altitude coastal zones showing signs of reduction. Under more severe climate scenarios, the expansion of the high‐suitability zone is more pronounced. However, concurrently, some medium‐suitability areas in central regions transition into low‐suitability zones, likely resulting from the combined effects of factors like climate warming. Under the low‐emission scenario (SSP1‐2.6), the total suitable habitat area generally increases, primarily driven by the expansion of medium‐ and low‐suitability zones. Under the medium‐emission scenarios (SSP2‐4.5, SSP3‐7.0), the total suitable habitat area continues to contract, indicating that persistent environmental pressures exert an inhibitory effect on species distribution. Under the high‐emission scenario (SSP5‐8.5), the total suitable habitat area increases during the 2050s and 2090s; by the 2090s, the high‐suitability zone area reaches 279,800 km^2^, the highest value observed across all scenarios. Concurrently, the proportion of high‐suitability zones increases to 20.1%, suggesting that climate change may drive the conversion of some low‐suitability areas into high‐suitability zones. Overall, the suitable habitat areas for *C. barometz* exhibit growth under low‐emission pathways, potential expansion under high‐emission pathways, while facing persistent decline risks under medium‐emission pathways. This pattern aligns with the observed characteristics of plant migration under current global warming trends (Zhang and Lin 2025).

**TABLE 2:** Area of national suitable habitats for *C. barometz* under different scenarios (×10^4^ km^2^).

Climate scenario	Period	Total suitable area	Low suitable area	Mod. suitable area	High suitable area
SSP1‐2.6	2030s	131.98	66.56	39.18	26.25
	2050s	128.24	70.88	35.66	21.69
	2070s	126.67	66.41	37.80	22.46
	2090s	147.33	78.88	42.69	25.76
SSP2‐4.5	2030s	141.05	76.18	41.96	22.91
	2050s	133.23	71.32	36.69	25.21
	2070s	128.68	69.81	35.32	23.54
	2090s	128.90	68.12	37.49	23.30
SSP3‐7.0	2030s	133.04	72.83	37.86	22.35
	2050s	128.78	66.11	39.03	23.64
	2070s	123.29	65.88	34.46	22.95
	2090s	130.49	71.24	35.65	23.61
SSP5‐8.5	2030s	129.49	66.28	37.71	25.50
	2050s	135.63	75.21	36.12	24.29
	2070s	125.24	66.40	35.47	23.38
	2090s	139.41	69.82	41.61	27.98

### 3.4 Centroid Migration Analysis of *C. barometz* Suitable Habitat Under Current and Future Climate

**FIGURE 6:**
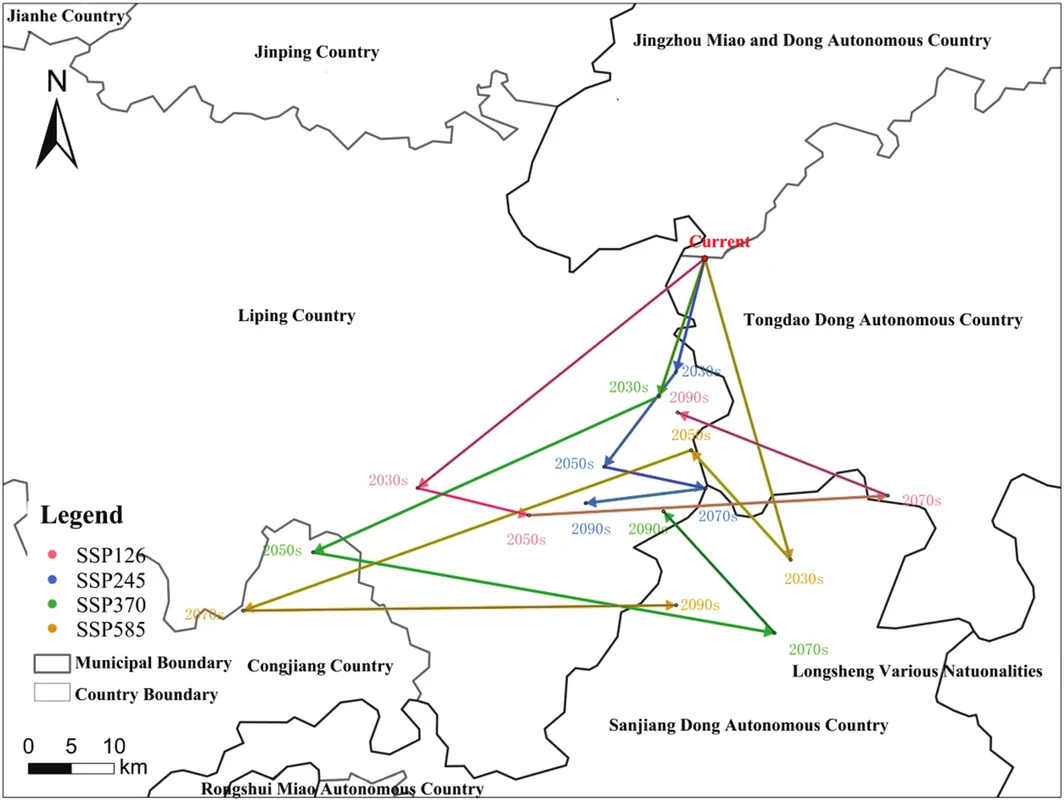
Centroid migration of the suitable habitat for *C. barometz*.

Under the future climate scenario, the centroid of the suitable area of *C. barometz* generally shows a migration trend from north to south (Figure 6). The centroid of the current suitable area is located in Tongdao Dong Autonomous County, Huaihua City, Hunan Province (109.47°E, 26.27°N). Under the low emission scenario (SSP1‐2.6), the mass center moves southward to Liping County, Guizhou Province (the total migration distance is 114.33 km). Under the high emission scenario (SSP5‐8.5), the centroid further moves southward to Sanjiang County, Guangxi (the total migration distance reached the farthest, 148.95 km). The specific migration path and coordinates are detailed in Table S1.

### 3.5 Analysis of Main Ecological Factors Affecting the Ecological Suitability of *C. barometz*

The results of the ecological factor contribution rates are shown in Table 3. The factors with significant contributions to the distribution of *C. barometz* are as follows: bio17 (49.9%), bio16 (9.1%), bio1 (5.1%), bio7 (4.2%), bio4 (3.6%), bio3 (2.8%), bio14 (2.7%), bio5 (2.4%), bio19 (1.9%), slo (1.9%), and bio15 (1.5%). The cumulative contribution rate of these factors is 85.1%.

**TABLE 3:** Contribution rates of main environmental factors affecting *C. barometz* distribution.

Ecological factor	Contribution%	Permutation importance	Ecological factor	Contribution%	Permutation importance
bio17	49.9	2.4	t_clay	1.1	4
bio16	9.1	4.8	t_caco3	1	2.6
bio1	5.1	7.9	t_cec_clay	0.9	2.3
bio7	4.2	15.3	bio2	0.8	2.8
bio4	3.6	5	t_gravel	0.8	1.4
bio3	2.8	2.9	bio13	0.7	2.2
bio14	2.7	4.4	t_usda_tex	0.6	2
bio5	2.4	5.1	t_teb	0.5	2
bio19	1.9	4.5	t_sand	0.5	1.8
slo	1.9	3.9	t_ref_bulk	0.4	0.7
bio15	1.5	2.3	t_silt	0.3	1.9
asp	1.4	0.8	t_oc	0.3	1.8
alt	1.3	7.2	t_ph_h2o	0.3	0.1
bio18	1.2	4.2	t_ece_soil	0.1	0.7
bio10	1.2	1.1	t_esp	0.1	0.1
bio8	1.1	2.1	t_ece	0	0

**FIGURE 7:**
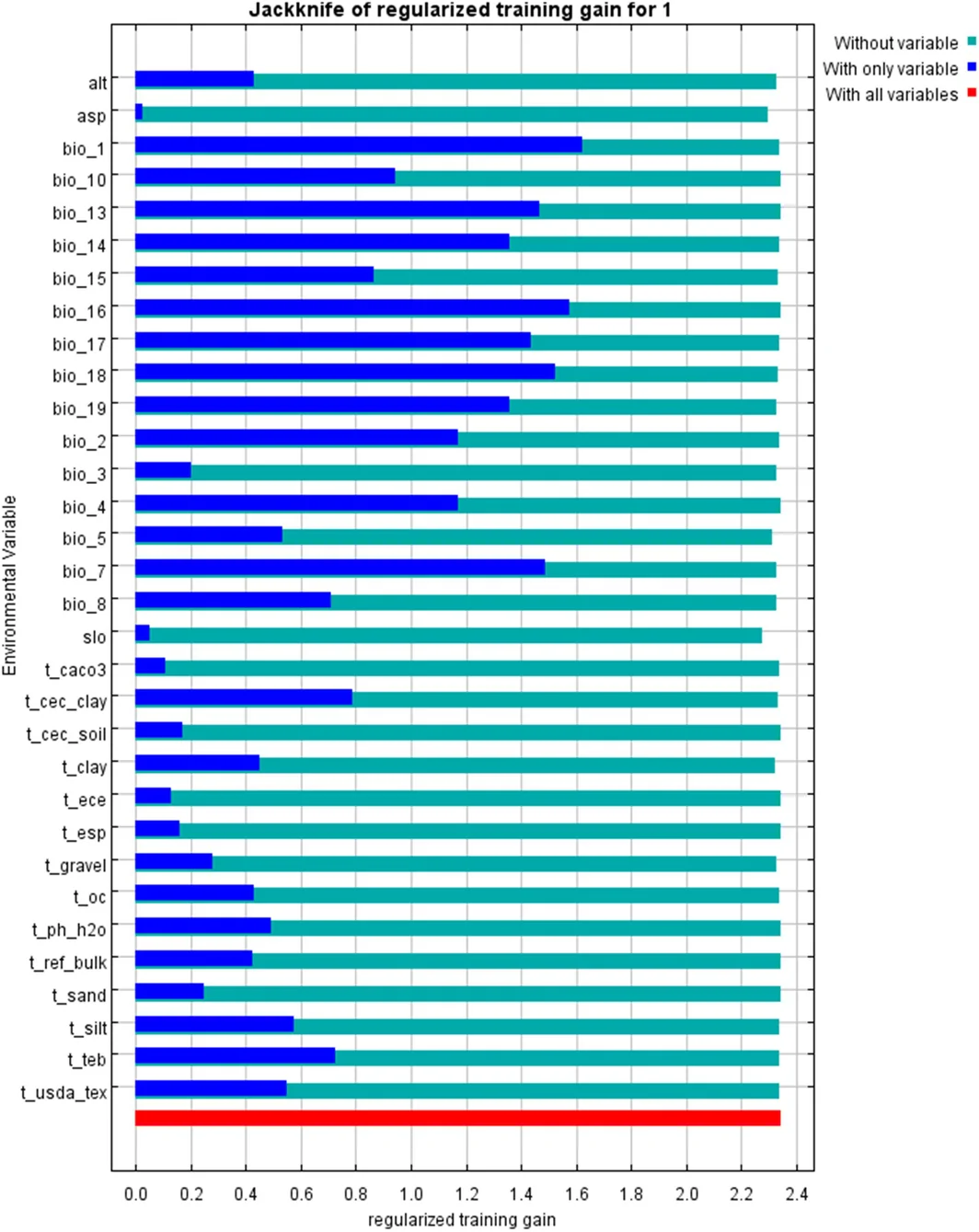
Jackknife analysis results of ecological factors for *C. barometz*.

**FIGURE 8:**
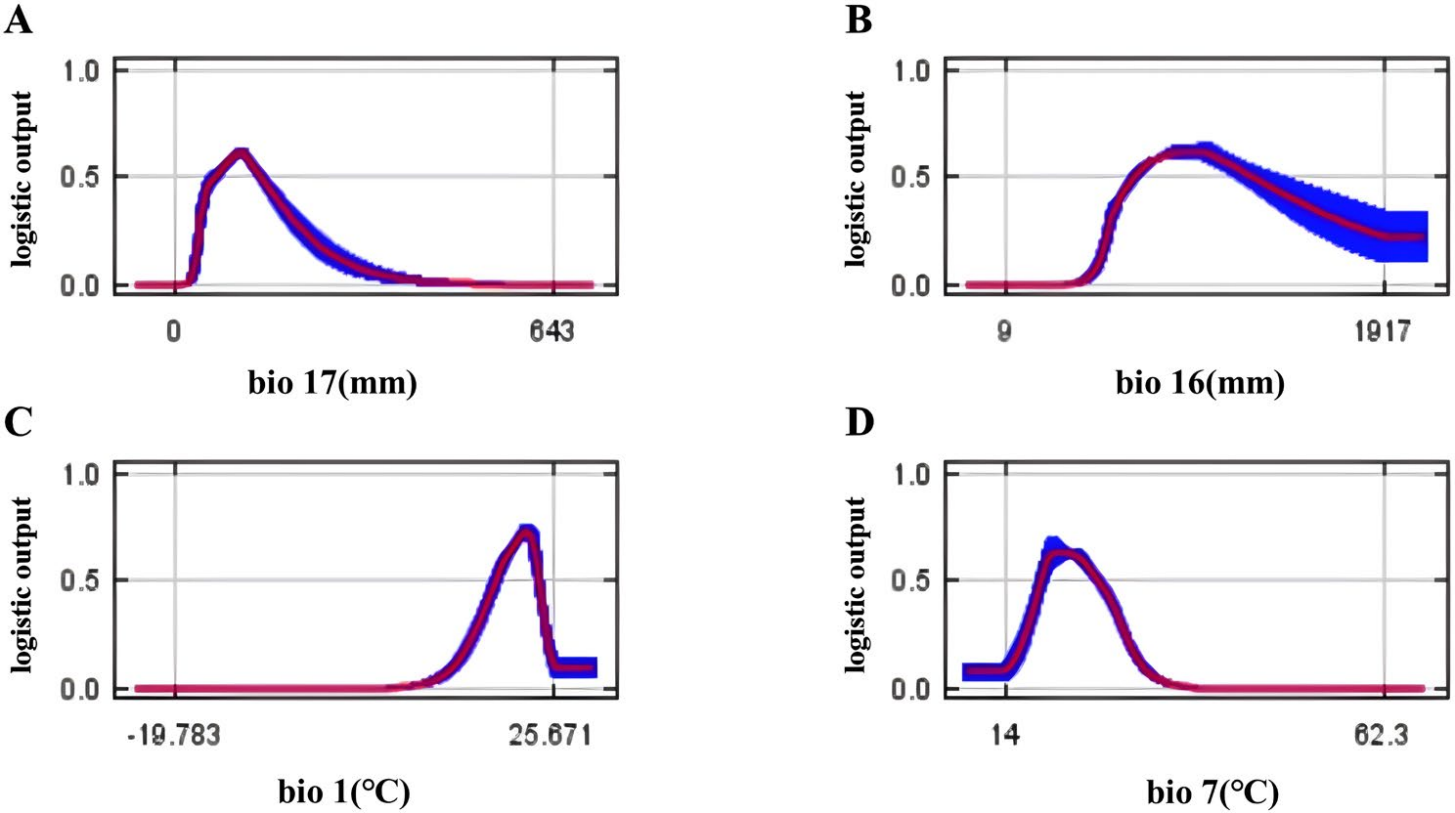
Response curves of main ecological factors. Arranged from highest to lowest contribution rate (based on Table 3): (A) bio17 (49.9%), (B) bio16 (9.1%), (C) bio1 (5.1%), (D) bio7 (4.2%).

The Jackknife test results (Figure 7) show that when using a single environmental variable, the six ecological factors with the greatest impact on normalized training gain are: bio1, bio16, bio18, bio7, bio13, and bio17. Following comprehensive analysis, bio17, bio16, bio1, and bio7 were ultimately selected for response curve analysis (Figure 8). The analysis predicts the optimal environmental parameters for the distribution of *C. barometz* as: Precipitation of Driest Quarter (bio17) between 50 and 150 mm, Precipitation of Wettest Quarter (bio16) between 300 and 1000 mm, Annual Mean Temperature (bio1) between 24°C and 26.5°C, and Temperature Annual Range (bio7) between 18°C and 25°C.

## 4 Discussion

### 4.1 Potential Suitable Distribution Areas of *C. barometz* in China

This study predicted the potential suitable areas for *C. barometz* based on 191 current occurrence records across China and 37 ecological factors. The MaxEnt model results were evaluated using the Receiver Operating Characteristic (ROC) curve, yielding a mean AUC value of 0.976, indicating that the predicted outcomes accurately reflect the potential suitable distribution of *C. barometz* in China. Under current climatic conditions, the highly suitable areas for *C. barometz* were mainly distributed in Guangdong, Guangxi, Guizhou, and Yunnan, which is consistent with the records in the *Flora of China*. Within Guizhou Province, the highly suitable areas were concentrated in the southern, southeastern, and southwestern regions. These areas represent highly developed karst landscapes in Guizhou; however, *C. barometz* does not grow directly on carbonate rocks. Instead, it is an indicator plant of acidic soils, strictly distributed in acidic soil types such as red soil and yellow soil (pH 4.5–6.5), with no distribution in pure calcareous soil areas. Taking Dushan County as an example, *C. barometz* is found only in low mountain valleys of non‐karst regions, where the soil is acidic yellow soil (Guizhou Forestry Bureau 2000). Therefore, the relationship between this species and “karst” is essentially one of spatial co‐occurrence rather than geological dependence: climatic factors determine the broad‐scale distribution pattern, while acidic soil patches act as a filtering mechanism at finer scales.

### 4.2 Key Ecological Factors Influencing the Potential Suitability of *C. barometz*

Our results indicate that under current climatic conditions, the primary ecological factors influencing the suitable distribution of *C. barometz* in China are precipitation of the driest quarter (bio17), precipitation of the wettest quarter (bio16), annual mean temperature (bio1), and temperature annual range (bio7), with a cumulative contribution rate of 68.3%. Notably, the timing of the driest quarter exhibits significant regional variation across China. In southwestern China (including Guizhou Province, the core area of this study), the driest quarter occurs mainly in winter (December to February), with some years experiencing winter–spring drought extending from October of the previous year to April–May of the following year (Qin et al. 2024). The optimal precipitation of the driest quarter for *C. barometz* ranges from 50 to 150 mm, which corresponds precisely to the winter dry season precipitation range in southwestern China, indicating that this species possesses a certain ecological adaptability to winter–spring drought. In contrast, spring drought (March to May) in northern China typically features precipitation below 50 mm, coinciding with the early growing season. This likely explains why *C. barometz* cannot expand northward—water deficit during the growing season severely limits establishment of young sporophytes and population maintenance (Zhou and Huang 2006; Wang et al. 2025). This finding is consistent with the species' biological requirement for humid conditions for spore reproduction (Pittermann et al. 2013). It is worth noting that different tree fern species exhibit markedly different responses to climatic limiting factors. A study on the Chinese tree fern *Alsophila costularis* found that temperature annual range (bio7) was the primary limiting factor for its distribution, rather than water‐related variables (Wang et al. 2024). This discrepancy reflects ecological strategy divergence between the two tree ferns: *A. costularis* is mainly limited by temperature seasonality, whereas *C. barometz* is more sensitive to dry season water availability, possibly due to its habitat characteristics of growing in acidic soil patches within karst regions where water supply during the dry season is limited. Second, annual mean temperature (bio1) restricts the distribution of *C. barometz* to a narrow range of 24°C–26.5°C. In high‐altitude, low‐temperature regions such as the Qinghai‐Tibet Plateau, the annual mean temperature is far below this threshold, failing to meet the thermal requirements for spore germination and gametophyte development, which explains the absence of this species in low‐temperature areas. Conversely, the mild winter climate (10°C–16°C) maintained by the Yunnan‐Guizhou quasi‐stationary front effectively mitigates these thermal risks (Cai et al. 2022). This topographic‐climatic coupling mechanism explains why southwestern Guizhou maintains thermal conditions suitable for *C. barometz* survival during winter. Furthermore, although slope contributes relatively little (1.9%) to the suitable habitat of *C. barometz* , gentle slopes of 15°–30° exhibit stronger soil water retention capacity compared to steep slopes, while also facilitating spore dispersal through surface runoff (Wang et al. 2022). These factors collectively contribute to the formation of highly suitable areas in southwestern Guizhou.

### 4.3 Changes in Potential Suitable Habitats of *C. barometz* Under Different Climate Scenarios

We conducted a centroid shift analysis to quantify the response rate of *C. barometz* to climate change and assess its vulnerability. The results indicate that the differences in suitable habitat area under different future climate emission scenarios reveal the complex impact of climate change on the distribution of *C. barometz* . Under the low‐emission pathway (SSP1‐2.6), the suitable habitat area shows an increasing trend. Under the medium‐emission pathways (SSP2‐4.5 and SSP3‐7.0), the suitable habitat area continuously declines, with low‐altitude areas in southern Guizhou potentially degrading into low‐suitability areas. Under the high‐emission pathway (SSP5‐8.5), the highly suitable area is projected to expand to 279,800 km^2^ by the 2090s, with its centroid shifting 148.95 km from Tongdao County in Hunan to Sanjiang County in Guangxi. This phenomenon is consistent with the climate refugia theory (Ashcroft 2010), suggesting that when the annual mean temperature in the northern part of the original habitat exceeds the critical threshold of 26.5°C, the species migrates toward low‐latitude mountainous areas to maintain hydrothermal balance. Cross‐species studies further support this interpretation. Karichu et al. (2024) found that most tree fern species in Africa persist under climate change precisely by maintaining their presence in refugial areas. Similarly, a genetic study on the Mexican tree fern *Alsophila firma* by Ramírez‐Barahona and Eguiarte (2014) demonstrated that areas with high environmental suitability exhibit significantly higher genetic diversity, which results from larger population sizes and greater inter‐population connectivity within refugia. In summary, Sanjiang County in Guangxi and the southern regions of Guizhou should be considered potential climate refugia for *C. barometz* under future climate warming. Priority conservation of these areas is crucial not only for the short‐term persistence of the species but also for maintaining its genetic diversity and long‐term evolutionary potential.

### 4.4 Implications for the Conservation Strategy of *C. barometz*

Based on the predicted results, implementing targeted conservation measures is essential. To this end, we propose a tiered conservation strategy. For areas with high suitability, such as southwestern Guizhou and southern Guangdong, establishing nature reserves is a key measure to limit anthropogenic disturbances, including harvesting and land reclamation, thereby protecting the evergreen broadleaf forests and shaded, humid ravine habitats on which *C. barometz* depends. Currently, Guizhou Province has a forest area of 11,133,300 ha, with a forest coverage rate of 63.3%. The ecological protection red line in the province covers no less than 40,800 km^2^ (Guizhou Forestry Bureau 2025; State Council of China 2023). Due to the strict dependence of *C. barometz* on shaded, humid understory environments and acidic soils, its potential available habitats are mainly located in the evergreen broadleaf forest regions of southern, southeastern, and southwestern Guizhou. Considering the distribution of forests, the ecological protection red line, and acidic soil patches, the available habitat area is conservatively estimated to be approximately 40,000–60,000 km^2^. We assessed the current coverage of *C. barometz* by existing protected areas in Guizhou Province (Table S2). Guizhou currently has 237 protected areas. Among these, the distribution area in Libo County has been incorporated into the Maolan National Nature Reserve; the distribution areas in Chishui and Xishui counties have been incorporated into the Chishui *Alsophila* National Nature Reserve; and Sandu County has established the country's first *C. barometz* ecological restoration base (Guizhou Forestry Bureau 2025; Sandu County Forestry Bureau 2024). Collectively, an estimated 60%–70% of known occurrence points are already within the coverage of the existing protected area network. However, conservation gaps remain in Luodian County, Wangmo County, Congjiang County, and Rongjiang County, and we recommend prioritizing these areas for inclusion in the protection system. For areas with conservation gaps and populations experiencing decline (e.g., central Guizhou), we recommend implementing ex situ conservation and establishing germplasm resource banks. Guanling County in Guizhou has successfully developed spore propagation technology for *C. barometz* , with an annual production capacity of 100 million plants, and has cultivated over 12 million mature plants (Anshun Forestry Bureau 2025). This successful experience can be extended to conservation gap areas by adopting a tripartite collaboration model involving enterprises, farmers, and research institutions. Under this model, understory cultivation can be carried out in low‐ to moderate‐suitability areas, providing a sustainable supply that reduces harvesting pressure on wild populations. In summary, this study proposes a synergistic development framework of “ecological protection—artificial propagation—community participation,” translating climate suitability predictions into actionable conservation actions and providing a scientific basis for the sustainable utilization of *C. barometz* and other endangered medicinal plants.

## 5 Conclusion

This study identifies the key environmental determinants of *C. barometz* distribution as precipitation extremes (driest and wettest quarters) and annual mean temperature, with precipitation of the driest quarter (bio17) as the dominant limiting factor. Under current climate conditions, the high‐suitability zone covers only 24,410 km^2^, primarily in Guangdong, Guangxi, and southwestern Guizhou. Future projections reveal divergent trajectories: the low‐emission pathway (SSP1‐2.6) supports habitat stability with moderate expansion, while the high‐emission pathway (SSP5‐8.5) projects expansion to 279,800 km^2^ by the 2090s, accompanied by a pronounced southward centroid shift of 148.95 km to Sanjiang County, Guangxi. In contrast, medium‐emission scenarios (SSP2‐4.5 and SSP3‐7.0) risk significant habitat contraction, with low‐elevation areas in southern Guizhou potentially degrading to low‐suitability zones. The consistent southward migration across scenarios indicates that *C. barometz* may retreat toward climate refugia as thermal thresholds are exceeded.

For conservation, we recommend: (1) prioritizing high‐suitability areas in the Guangdong‐Guangxi‐Guizhou corridor for in situ protection; (2) promoting wild‐simulated understory cultivation in medium‐suitability areas; (3) establishing ex‐situ conservation in central Guizhou where habitat decline is projected. This integrated approach aligns ecological conservation with sustainable use in this climate‐sensitive biodiversity hotspot.

## Author Contributions

**Fulin Yan:** conceptualization (equal), data curation (equal), formal analysis (equal), funding acquisition (equal), investigation (equal), methodology (equal), resources (equal), writing – original draft (equal), writing – review and editing (equal). **Songsong Wu:** formal analysis (equal), validation (equal). **Wenxi Xiang:** formal analysis (equal), validation (equal), visualization (equal). **Qingwen Sun:** validation (equal). **Shenghua Wei:** conceptualization (equal), funding acquisition (equal), project administration (equal), resources (equal), supervision (equal), validation (equal), writing – review and editing (equal).

## Funding

This work was supported by National Administration of Traditional Chinese Medicine: National Base Construction Project for Seed and Seedling Breeding of Basic Traditional Chinese Medicine Materials (Guizhou), Contract: 2014–2017. Guizhou Provincial Forestry Bureau 2026 Key Research Project: Research on in‐forest Cultivation Techniques for Traditional Chinese Medicinal Herbs, Qian‐Lin‐Ke‐He [2026] Key 006. Guizhou Provincial Department of Agriculture and Rural Affairs: Construction of Guizhou Provincial Modern Industrial Technology System for Chinese Medicinal Materials, GZCYTX 2026‐0203. Project of Guizhou Provincial Administration of Traditional Chinese Medicine, QZZY‐2026‐083.

## Conflicts of Interest

The authors declare no conflicts of interest.

## Supporting information


**Table S1:** Overview of centroid migration for *C. barometz* suitable habitat.
**Table S2:** Conservation status of C. barometz in Guizhou.

## Data Availability

All necessary data have already been provided in the main text and Supporting Information.
