# Supplementary material for: The Current and Future Distribution Areas of the Rare and Endangered Fern Cibotium barometz (Cibotiaceae) in Guizhou, China, Based on the MaxEnt Model and ArcGIS
Source: Ecol Evol. 2026 Aug 26;16(9):e74203. doi: 10.1002/ece3.74203 (PMC13519528; doi:10.1002/ece3.74203)
Supplement: Supplementary file 1 [file ECE3-16-e74203-s001.docx]

Supplementary Materials

**Table S1 Overview of centroid migration for *C. barometz* suitable habitat**

| Scenario | Period | Direction | Distance (km) | Centroid Location |
| --- | --- | --- | --- | --- |
| - | Current | - | - | 109.47°E，26.27°N  Tongdao County, Huaihua City, Hunan Province |
| SSP1-2.6 | 2030s | Southwest | 40.28 | 109.17°E，26.03°N  Liping County, Qiandongnan Prefecture, Guizhou Province |
|  | 2050s | Southeast | 12.13 | 109.17°E，26.00°N  Liping County, Qiandongnan Prefecture, Guizhou Province |
|  | 2070s | Southeast | 37.81 | 109.67°E，26.02°N  Tongdao County, Huaihua City, Hunan Province |
|  | 2090s | Northwest | 24.11 | 109.45°E，26.11°N  Liping County, Qiandongnan Prefecture, Guizhou Province |
| SSP2-4.5 | 2030s | Southwest | 13.52 | 109.45°E，26.15°N  Liping County, Qiandongnan Prefecture, Guizhou Province |
|  | 2050s | Southwest | 13.37 | 109.37°E，26.05°N  Liping County, Qiandongnan Prefecture, Guizhou Province |
|  | 2070s | Southeast | 10.97 | 109.47°E，26.03°N  Liping County, Qiandongnan Prefecture, Guizhou Province |
|  | 2090s | Southwest | 12.73 | 109.35°E，26.01°N  Liping County, Qiandongnan Prefecture, Guizhou Province |
| SSP3-7.0 | 2030s | Southwest | 16.73 | 109.43°E，26.12°N  Liping County, Qiandongnan Prefecture, Guizhou Province |
|  | 2050s | Southwest | 40.65 | 109.07°E，25.96°N  Congjiang County, Qiandongnan Prefecture, Guizhou Province |
|  | 2070s | Southeast | 49.47 | 109.55°E，25.88°N  Sanjiang County, Liuzhou City, Guangxi Zhuang Autonomous Region |
|  | 2090s | Northwest | 18.35 | 109.43°E，26.01°N  Liping County, Qiandongnan Prefecture, Guizhou Province |
| SSP5-8.5 | 2030s | Southeast | 36.18 | 109.57°E，25.96°N  Sanjiang County, Liuzhou City, Guangxi Zhuang Autonomous Region |
|  | 2050s | Northwest | 16.50 | 109.46°E，26.07°N  Tongdao County, Huaihua City, Hunan Province |
|  | 2070s | Southwest | 50.68 | 108.99°E，25.90°N  Congjiang County, Qiandongnan Prefecture, Guizhou Province |
|  | 2090s | Southeast | 45.59 | 109.45°E，25.91°N  Sanjiang County, Liuzhou City, Guangxi Zhuang Autonomous Region |

**Table S2 Conservation status of *C. barometz* in Guizhou**

| County/District | Predicting the grade of suitable area | Covered protected areas | Conservation status | Recommended actions |
| --- | --- | --- | --- | --- |
| Libo County | Moderate | Maolan National Nature Reserve | Protected | Strengthen management |
| Leishan County | Low | Leigongshan Nature Reserve | Protected | Strengthen management |
| Chishui City | High | Chishui *Alsophila* National Nature Reserve | Protected | Strengthen management |
| Xishui County | Low | Chishui *Alsophila* National Nature Reserve | Protected | Strengthen management |
| Sandu County | High | *C. barometz* Ecological Restoration Base | Protected | Expand scale |
| Congjiang County | High | None | Conservation gap | Prioritize for inclusion in protection system |
| Rongjiang County | High | None | Conservation gap | Prioritize for inclusion in protection system |
| Luodian County | High | None | Conservation gap | Prioritize for inclusion in protection system |
| Wangmo County | High | None | Conservation gap | Prioritize for inclusion in protection system |
